# Long-Term Survival of Patients with Mantle Cell Lymphoma after Total Body Irradiation, High-Dose Chemotherapy and Stem Cell Transplantation: A Monocenter Study

**DOI:** 10.3390/cancers15030983

**Published:** 2023-02-03

**Authors:** Kai Kröger, Jan Siats, Andrea Kerkhoff, Georg Lenz, Matthias Stelljes, Hans Theodor Eich, Gabriele Reinartz

**Affiliations:** 1 Department of Radiation Oncology, University Hospital of Muenster, Albert-Schweitzer Campus 1, Building 1A, 48419 Muenster, Germany; 2 Bone Marrow Transplantation Unit, Department of Hematology and Oncology, University Hospital of Muenster, Albert-Schweitzer Campus 1, Building 1A, 48419 Muenster, Germany

**Keywords:** mantle cell lymphoma, total body irradiation, stem cell transplantation

## Abstract

**Simple Summary:**

Mantle cell lymphoma (MCL) is a very rare subtype of non-Hodgkin‘s lymphoma, predominantly affecting men, where most patients at initial presentation show an advanced spread of the disease with poor prognosis. We analyzed the long-term outcome of 22 patients treated with total body irradiation (TBI)-based conditioning prior to stem cell transplantation (SCT) at the University Hospital of Muenster, Germany. The present results of multimodal treatment support the published reports that TBI-based high-dose therapy followed by SCT is highly effective in this unfavorable disease. Both recently approved agents and traditional SCT open up possibilities of a personalized treatment approach for MCL with the aim of a cure.

**Abstract:**

Introduction: In patients with mantle cell lymphoma (MCL), long-term remissions can be achieved by stem cell transplantation (SCT). Different conditioning treatment protocols exist with or without total body irradiation (TBI). There are few data published on the role of TBI before autologous stem cell transplantation (autoSCT) or allogenic stem cell transplantation (alloSCT). We report on the long-term survival data of patients treated by TBI prior to autologous or allogenic SCT at our center. Patients: In a retrospective analysis, the data of patients treated at the University Hospital of Muenster from May 2004 to February 2015 were collected and evaluated. For the analysis, all data of patients who were histopathologically diagnosed with MCL and underwent TBI prior to stem cell transplantation (SCT) were evaluated. Results: A total of 22 patients (19 men and 3 women) were treated with a TBI-based conditioning prior to SCT. The median age at initial diagnosis was 57.5 years (38–65 years). Seventeen patients had Ann Arbor stage IV, two patients had Ann Arbor stage III, and three patients Ann Arbor stage II disease. AutoSCT was performed in 19 patients and alloSCT was performed in 3 patients. In 18 patients, autoSCT was applied as part of first-line therapy, and in one patient after relapse. Two patients received alloSCT after relapse of MCL, and one patient received alloSCT during first-line therapy after an inadequate treatment response. TBI was performed in 12 patients with 10 Gy and in 6 patients with 12 Gy, these patients subsequently received autoSCT. In the group of four patients who received TBI with four Gy, four patients subsequently received alloSCT and one patient received autoSCT. Median overall survival after autoSCT and previous TBI was 11.4 years (142 months). In total, 11 out of 19 patients treated with autoSCT lived longer than 6.8 years (82–202 months). After alloSCT and previous TBI, the median overall survival was 3.25 years (14–59 months). Conclusions: A large proportion of patients with advanced MCL survived remarkably longer than 11.4 years after high-dose chemotherapy, TBI, and SCT. The present results of multimodal treatment support the published reports that TBI-based high-dose therapy followed by autoSCT is highly effective in this prognostically unfavorable disease situation.

## 1. Introduction

Mantle cell lymphoma (MCL) is a rare subtype of non-Hodgkin’s lymphoma (NHL) that accounts for approximately 6% of all NHLs around the world (International Lymphoma Study Group 1997) and 6–8% of all NHLs in Europe [1]. MCL is characterized by a high expression of cyclin D1 caused by the chromosomal translocation t(11;14). The annual incidence of MCL has increased to 1–2 per 100,000 persons at risk. The male/female ratio of the disease is 3:1. According to the Surveillance, Epidemiology and End Results (SEER) database, trends in incidence of MCL by age and by tumor stage are shown: the median age at the time of MCL diagnosis was 68 years and the age-adjusted incidence rate for stage IV increased dramatically over time with an annual raise of 10.22% [2].

Mantle cell lymphoma is characterized by poor outcome, but with the development of novel therapeutic agents and of modern radiation techniques, substantial progress has been made in the last years [3,4]. Mantle cell lymphomas are differentiated into subtypes behaving in an indolent or aggressive manner depending also on mutation profile and level of serum lactate dehydrogenase. Disease manifestations of mantle cell lymphoma can appear to be very heterogeneous. Most patients at initial presentation show an advanced spread of the disease requiring intensive systemic treatment. For patients up until the age of 65, aggressive immune-chemotherapy followed by autologous stem cell transplantation and maintenance therapy represents a standard of care [5].

In this study, we discuss the total body irradiation (TBI) conditioning prior to SCT concept at our University Hospital and newly approved treatment approaches.

## 2. Methods

We performed a retrospective analysis to evaluate the role of total body irradiation (TBI) in the conditioning regimen of autologous stem cell transplantation (autoSCT) and allogenic stem cell transplantation (alloSCT) in mantle cell lymphoma (MCL). Data were collected from May 2004 until February 2015 at the University Hospital of Muenster, Germany. The multimodal treatment was carried out according to interdisciplinary tumor board recommendation. The patients received total body irradiation in the Department of Radiation Oncology followed by stem cell transplantation at the Department of Hematology and Oncology. TBI was performed with the patient lying down in a bed and rotating in four positions shielded by 2 cm of acrylic glass to ensure adequate surface dosage. The bed is specially modified for TBI and is located at a distance of 545 cm from the focus of the linear accelerator. The total body irradiation was applied after computertomography-based three dimensional (3D) planning with 15 MV photons of a linear accelerator. An accompanying evaluation of dosimetry on patients was performed using diode system with detectors glued to the skin in eight positions (head, larynx, neck, mediastinum, left and right thorax, left and right abdomen) to secure the correct application of radiation dose. A possible adjustment of the monitor units based on the measured values is agreed between treating physician and medical physicist. TBI with a total dose of more than 8 Gy was performed with individual lung shielding to ensure effective protection against acute or chronic side effects. If patients were treated with lung shielding, they received individually adapted radiation of mediastinal and axillary lymph nodes as compensation. For mediastinal and axillary radiotherapy, 3D-conformal radiotherapy (3D-CRT) with 6 and 15 MV photons was applied. Daily online imaging verifications are performed before each radiation fraction for precise positioning control of patients. Total body irradiation, high-dose chemotherapy and stem cell transplantation are performed under inpatient treatment at the Bone Marrow Transplantation Unit, University Hospital of Muenster.

### Statistical Analysis

Overall survival (OS) was calculated using the Kaplan and Meier estimator. Survival was calculated from the time of diagnosis to death from any cause using data from national death records.

## 3. Results

### 3.1. Radiation Therapy

Myeloablative TBI was performed with 2 Gy fractions twice daily with linear accelerator-based 15 MV photons. A total of 18 patients were treated with lung blocks to limit the lung dose to 8 Gy; additional ap-pa photon fields were applied to the corresponding mediastinum and axillary region.

### 3.2. Patients

Overall, 22 patients (19 male and 3 female) with a mean age of 57.5 years (range 38–65 years) were eligible for this study. At diagnosis, seventeen patients presented with Ann Arbor stage IV, two patients presented with Ann Arbor stage III, and three patients presented with Ann Arbor stage II.

Nineteen patients underwent autoSCT and three patients underwent alloSCT. A total of 18 patients received autoSCT as first-line therapy. Only one patient received autoSCT after relapse. alloSCT was performed after relapse in two patients and in first-line therapy in one patient because autoSCT was not feasible. The patient information is summarized in Table 1. The detailed patient characteristics are shown in Table 2.

Eleven patients received chemotherapy consisting of three courses of (R)-CHOP and three courses of R-DHAP. One patient received two courses of CHOP and two courses of DHAP without rituximab due to severe respiratory insufficiency; four patients received six courses of R-CHOP, two patients received Rituximab and bendamustine (one of them with an additional four cycles of R-CHOEP and three cycles R-DHAP); three patients received different variations of chemotherapy with rituximab.

Twelve patients were conditioned with 10 Gy TBI and six patients were conditioned with 12 Gy TBI over three days with lung shielding. The chemotherapeutic conditioning varied between patients.

Twelve patients received cytarabine and melphalan, two patients received cytarabine, five received fludarabine and cyclophosphamide, and three received cyclophosphamide, respectively.

Four patients were conditioned with 4 Gy TBI on one day. This group comprised three patients receiving alloSCT and one patient receiving autoSCT.

It revealed no toxicity related to TBI conditioning. In particular, no cases of pneumonitis in the peri-transplant period have been documented. In the cohort, eleven deaths occurred: two patients died of progressive disease and three patients died due to therapy-induced sepsis (two patients underwent autoSCT and one alloSCT). Two out of these three septic patients were diagnosed with acute graft versus host disease. Six deaths occurred without a known link to MCL or therapy.

Median overall survival was 6.6 years (80 months) in the whole cohort. Regarding the sub-cohorts comprising patients treated with either autoSCT or alloSCT, the corresponding median overall survival was 11.4 years (142 months, range 7–207 months) and 3.25 years (14–59 months), respectively.

**Figure 1:**
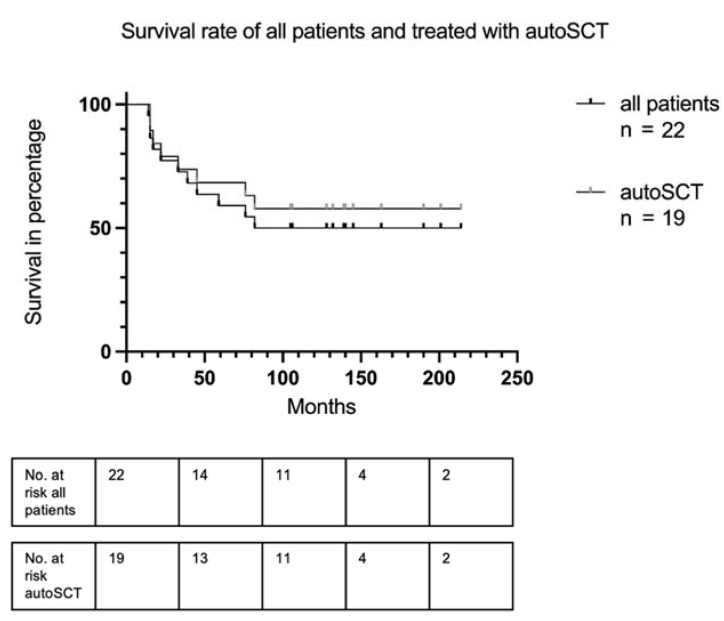
Overall survival of all patients and of patients treated with autoSCT.

Compared with the whole cohort, the 19 patients following autoSCT presented a better overall survival rate after reaching a plateau after 6.8 years (57.9 percent vs. 50.0 percent). The survival data are depicted in detail as a Kaplan–Meier plot in Figure 1. Data from 10 of the 19 patients with autoSCT were available regarding relapse details. Among these, the median duration of response was 18 months (5–127 months) (Table 1).

## 4. Discussion

Mantle cell lymphoma is mostly diagnosed in an advanced stage of disease and shows a heterogeneous and mainly aggressive course. The median survival is roughly 5 years, but a small portion of patients shows an indolent course of disease. Still, long-term survival remains a rarity and reliable prognostic markers are uncertain to prospectively identify the patients with an indolent course of disease [6,7].

Young and fit patients are commonly treated by aggressive chemotherapy in combination with rituximab and autologous stem cell transplantation [8,9,10,11]. A randomized phase 3 study of the European Mantle Cell Lymphoma Network led to the recommendation to add high-dose cytarabine to the immunochemotherapy before autoSCT [12]. The standard of care (SOC) for young and fit MCL patients is an immunochemotherapy, most frequently so-called BEAM, followed by autoSCT and Rituximab maintenance [13]. The traditional autoSCT with the purpose of prolonging remission is also evolving. Studies from France and North America found a remarkable improvement in PFS when autoSCT is followed by rituximab maintenance. Furthermore, overall survival is significantly improved [11]. For young and fit SCT-eligible patients who achieve a complete or partial response, high-dose chemotherapy with or without autoSC rescue still remains a trending option [14]. AutoSCT is the current standard therapy in fit patients with MCL in first remission. A European multicenter randomized phase 3 trial demonstrated that consolidation by myeloablative radiochemotherapy (TBI with 12 Gy) before autoSCT in first remission significantly prolonged PFS (3.3 vs. 1.5 years) and OS (7.5 vs. 4.8 years) in patients with primary advanced stage III-IV MCL [15].

Of several molecular-targeted therapies, Bruton’s tyrosine kinase inhibitor ibrutinib resulted in the highest response rates. The current review of Pu et al. shows the impressive number of recently approved agents for the treatment of MCL: lenalidomide (immunomodulatory drug), bortezomib (proteasome inhibitor) and ibrutinib, acalabrutinib, and zanubrutinib (Bruton’s tyrosine kinase inhibitors). The European Commission has approved lenalidomide, ibrutinib, and also temsirolimus (mTOR inhibitor), and for the treatment of mantle cell lymphoma, the other agents aforementioned have not yet been approved for MCL in the European Union. Epigenetic agents, mammalian target of rapamycin (mTOR) inhibitors, and monoclonal antibodies/antibody-drug conjugates (obinutuzumab, polatuzumab, and ublituximab) are promising therapeutic agents under clinical trial investigation.

It remains to be seen whether and in which patients targeted therapy with Bruton’s tyrosine kinase (BTK) inhibitors may be able to replace autologous stem cell transplantation in the future. In this respect, the longer-term results of the international triangle study, a randomized European MCL network trial, will be indicative [16].

Patients with relapsed/refractory (R/R) MCL and disease progression after Bruton’s tyrosine kinase inhibitor treatment have a poor prognosis. The anti-CD19 chimeric antigen receptor (CAR)-T-cell therapy (Tecartus) may induce remissions in patients with R/R MCL after failure of BTK inhibitor treatment [17]. Recently, the chimeric antigen receptor (CAR)-T cell therapy and bispecific T-cell engager (BiTE) therapy have been opening a new perspective for MCL treatment. Beyond these, investigational therapies exist including zilovertamab vedotin (an antibody-drug conjugate that targets ROR1) [18] and the noncovalent Bruton’s tyrosine kinase inhibitor pirtobrutinib [19]. Due to MCL’s complicated pathology and high relapse incidence, there still needs to be optimal therapeutic strategies for both first-line and relapsed/refractory disease.

Older, unfit patients are treated with less intense chemotherapy protocols and maintenance rituximab. In case of relapse, different lines of chemotherapy exist and in case of chemotherapy, refractory disease treatment options such as ibrutinib and venetoclax (not yet approved) exist [20,21,22].

TBI has been used to treat malignant hematopoietic and lymphoid tumors for more than a hundred years [23]. Despite showing some clinical responses, the usage of TBI as a single modality is obsolete because of the possible acute and chronic toxicities. Today, patients solely undergo TBI as a part of the conditioning for hematopoietic stem cell transplant (HSCT). 

The first report of the use of bone marrow transplantation after TBI or chemotherapy was published in 1957 by Donnall Thomas [24]. Since then, different HSCT treatment regimens mainly regarding alloSCT with or without TBI have been developed and HSCT is part of varying treatment protocols for MCL and other hematologic and lymphoid diseases in adults and children [8,25,26,27,28,29,30,31].

In our cohort, 8 of the 18 patients treated with TBI and autoSCT after the primary diagnosis of MCL lived longer than five years with a median time of survival of 11.4 years (82–202 months) after autoSCT and TBI.

Three patients receiving TBI as part of an allogenic SCT showed a median survival of 3.25 years (14–59 months). This limited outcome can be attributed to the fact that two of the patients received alloSCT in relapsed disease and one as part of the primary treatment regime after showing a persisting bone marrow involvement after induction chemotherapy. However, one patient (19) treated with autoSCT at first relapse underwent salvage alloSCT 22 months after autoSCT due to second relapse. He is still alive more than 7 years after alloSCT.

The currently used influencing factors for prognostication of MCL are the MCL international prognostic index (MIPI), Ki-67 proliferation index, and TP53 mutation status. To address the question why some patients have profited more than others, we evaluated the risk score MIPI and if the Ki-67 proliferative index was available the c-MIPI as well as possible p53-alterations.

The majority of patients were diagnosed with MCL more than 10 years ago and the risk factors and scores were not established in clinical routine at that time. MIPI was calculable in 9 of 22 patients, and the initial Ki-67 index was available in one patient. A p53 mutation was diagnosed in one patient.

Our data show that a large cohort of patients with advanced MCL can profit from alloSCT or autoSCT including TBI. However, the small cohort, and the retrospective and monocentric character of this study mean several limitations. No specific group of patients profiting from stem cell transplantation can be defined as no control group exists and no data regarding risk factors were available.

## 5. Conclusions

We conclude that autoSCT including TBI is an established and effective treatment modality at our University Hospital and gives patients with rare MCL an opportunity for long-term survival. The heterogeneity in MCL pathology, the unpredictability in prognosis, and the variability in treatment response moves MCL into the focus of new therapy development. The aim will be to develop a better understanding of these trends and to issue a prospective view on optimizing individual treatment concepts.

Prospective studies applying the interdisciplinary multimodal treatment followed by standardized follow-up evaluation could be of use. The Ki-67 proliferation index and the p53 mutation status as well as the LDH level are essential criteria for the implementation of the therapy concept. Mutation analysis in addition to primary diagnostics should be set in correlation with treatment response for further improvement and individualization of the treatment approach.

Immunochemotherapy treatment followed by autoSCT and CAR T-cell constructs may have the potential to cure MCL patients. Nevertheless, after both autoSCT and CAR T-cell therapy, serious and life-threatening toxic events were reported [12,17]. Unfortunately, many MCL patients relapse, even after a high-dose consolidation regimen. For less fit patients, the option of myeloablative radiochemotherapy followed by autoSCT could be considered.

The intended goal is to develop personalized combination treatment approaches adapted to patient age and lymphoma aggressiveness to cure this disease.

## Figures and Tables

**Table 1:** Summarized patient characteristics.

Parameter	No.
	22
Age (year) (median)	38–65 (57.5)
Sex	
Male	19
Female	3
Ann Arbor stage	
II	3
III	2
IV	17
Stem cell transplantation	
autoSCT	19
alloSCT	3
Total body irradiation (total dose)	
12 Gy	6
10 Gy	12
4 Gy	4
Follow-up time (months)	14–214 (median 94)
Duration of response (months) *	5–127 (18)

* Duration of response in 10/19 patients with autoSCT.

**Table 2:** Detailed patient characteristics.

Age at Diagnosis	Stage	Nodal Manifestation	Bone Marrow Involved	SCT Type	Chemotherapy	Conditioning Regimen	TBI-Dose (Gy)	Relapse Details	Time of First Relapse (months)	Survival Since TBI (months)	Still Alive	MIPI	Details
43	IV A	No	Yes	Autologous	3x R-CHOP und 3x R-DHAP	Cytarabine melphalan	10	CR since 10/2010		137.00	Yes	Low	TP 53 aberration, in remission
59	IV B	Multilocular	Yes	Autologous	3x R-CHOP und 3x R-DHAP	Cytarabine melphalan	10	Relapse after 96 months, Ibrutinib started	96	135.00	Yes	Low	SD
59	IV B	Axillary and mediastinum	Yes	Autologous	3x R-CHOP und 3x R-DHAP	Cytarabine melphalan	10	None or n.a.		12.00	No	N.a.	Unknown
61	IV S B	Multilocular	Yes	Autologous	6x R-CHOP 21	Cytarabine melphalan	10	PD after 13 months, start R-DHAP	13	8.00	No	Low	Death due to sepsis during chemotherapy
50	IV B	Multilocular	Yes	Autologous	2x CHOP und 2x DHAP	Cytarabine	10	PD bone marrow after 12 months, start Velcade/Dexamethason	12	15.00	No	N.a.	Died of pulmonary embolism
57	IV A	Multilocular	Yes	Autologous	6x R-CHOP	Cytarabine	12	PD after 67 months, start R-DHAP; next PD 60 months later, local radiotherapy 39.6 Gy	67	207.00	Yes	N.a.	CR
41	IV A	Multilocular	No	Autologous	3x R-CHOP und 3x R-DHAP	Cytarabine melphalan	10	PD after 20 months nodal + bone marrow; start chemotherapy (not specified)	20	26.00	No	N.a.	Died of PD (meningeosis)
64	IV	Multilocular	Yes	Autologous	2x BR und 4x Bendamustin	Fludarabine, cyclophosphamide, ATG	4	Chronic GvHD		56.00	No	N.a.	Died of infection and myocardial ischemia
60	IV A	Multilocular	Yes	Autologous	3x R-CHOP und 3x R-DHAP	Cytarabine melphalan	10	Uncertain PD in PET CT after 10 months, local radiotherapy 30.6 Gy	10	184.00	Yes	N.a.	Remission
58	II	N.a.	N.a.	Autologous			10	None or n.a.		39.00	No	N.a.	Unknown
50	II A	Neck	No	Autologous	3x R-CHOP und 3x R-DHAP	Cytarabine melphalan	10	None or n.a.		100.00	Yes	N.a.	Unknown
53	IV A	Multilocular	Yes	Autologous	2x CHOP und 4x R-CHOP	Fludarabine, cyclophosphamide	12	Residual activity after 5 months	5	7.00	No	Intermediate	Died of acute Gvhd
63	IV B E	Multilocular	N.a.	Autologous	3x R-CHOP und 3x R-DHAP	Cytarabine	10	PD after 127 months; bulk axillary, treatmet not specified	127	158.00	Yes	Intermediate	PR, receiving ibrutinib + rituximab
51	IV	Multilocular	Yes	Autologous	3x R-CHOP und 3x DHAP	Cytarabine melphalan	10	None or n.a.		134.00	Yes	High	Unknown
43	IV A	Multilocular	Yes	Autologous	6 x R-CHOP 21	Cyclophosphamide	12	Secondary acute myeloic leukemia 11/2011		59.00	No	Low	Died due to multi organ failure
38	III	N.a.	N.a.	Autologous	6x R-CHOP	Cyclophosphamide	12	None or n.a.		194.00	Yes	N.a.	unknown
54	IV A S	No	Yes	Autologous	3x R-CHOP und 3x R-DHAP	Cytarabine melphalan	10	None or n.a.		100.00	Yes	N.a.	Unknown
38	III A	Multilocular	N.a.	Autologous/allogeneic		Cyclophosphamide	12	PD after 16 months, next PD 20 months later	16	110.00	Yes	N.a.	Remission
61	IV E	Multilocular	Yes	Autologous	4x R-CHOP-21-, 2x R-DHAP, 2x R-Dexa-BEAM	Cytarabine melphalan	12	PD after 72 months	72	120.00	Yes	N.a.	SD, receiving therapy
60	IV A	Multilocular	Yes	Allogeneic	8x BR, 4x R-CHOEP, 3x R-DHAP	Fludarabine, cyclophosphamide	4	PD after 30 months	30	3.00	No	N.a.	Died of sepsis, GvHD
65	II A	Neck	No	Allogeneic	4x Gemcitabine/Oxaliplatin und Rituximab (GEMOX-R)	Fludarabine, cyclophosphamide, ATG	4	PD 25 months, PD 8 months, PD 14 months	25	9.00	No	Low	Unknown
60	IV A	Multilocular	Yes	Allogeneic	3x R-CHOP und 3x R-DHAP	Fludarabine, cyclophosphamide	4	Acute GvHD		7.00	No	High	Died of complications after SCT

## Data Availability

The data presented in this study are available on request from the corresponding author.
